# Hog1p activation by marasmic acid through inhibition of the histidine kinase Sln1p

**DOI:** 10.1002/ps.4257

**Published:** 2016-03-22

**Authors:** Stefan Jacob, Anja Schüffler, Eckhard Thines

**Affiliations:** ^1^ Institut für Biotechnologie und Wirkstoff‐Forschung gGmbH (IBWF)KaiserslauternGermany; ^2^ Institut für Mikrobiologie und WeinforschungJohannes Gutenberg‐University MainzMainzGermany

**Keywords:** HOG pathway, sesquiterpenoids, osmoregulation, Magnaporthe oryzae, marasmic acid, sln1, histidine kinase, hik1

## Abstract

**BACKGROUND:**

The histidine kinase (HK) MoHik1p within the high‐osmolarity glycerol (HOG) pathway is known to be the target of the fungicide fludioxonil. Treatment of the fungus with fludioxonil causes an uncontrolled hyperactivation of the pathway and cell death. In this study, we used a target‐based in vivo test system with mutant strains of the rice blast fungus Magnaporthe oryzae to search for new fungicidal compounds having various target locations within the HOG pathway. Mutants with inactivated HOG signalling are resistant to fungicides having the target located in the HOG pathway.

**RESULTS:**

The HK MoSln1p was identified as being involved in the new antifungal mode of action of marasmic acid, as single inactivation of the genes MoSLN1, MoSSK1, MoSSK2, MoPBS2 and MoHOG1 resulted in mutant strains resistant against the sesquiterpenoid, whereas the wild‐type strain and the ΔMohik1 mutant were susceptible. Western blot analysis of phosphorylated MoHog1p confirmed the hypothesis that marasmic acid interferes with the HOG pathway, as a strong phosphorylation of MoHog1p was detectable after sesquiterpenoid treatment in the wild‐type strain but not in the ΔMosln1 mutant.

**CONCLUSION:**

This study provides evidence for marasmic acid activating the HOG pathway via the HK MoSln1p, and we propose that the sesquiterpenoid has a new mode of action in M. oryzae that differs from that of known HOG inhibitors, e.g. fludioxonil. © 2016 The Authors. *Pest Management Science* published by John Wiley & Sons Ltd on behalf of Society of Chemical Industry.

## 1 INTRODUCTION

Crop protection against phytopathogenic fungi has been progressively focused on a rational use of pesticide chemicals in which consumer health and environmental preservation dominate the entire developmental process. Sustainable fungicides are a key part in the modern control of crop plant diseases. For many decades, multisite contact fungicides were the only chemicals for the sufficient control of plant‐pathogenic fungi. The introduction of single‐site fungicides has revolutionised chemical plant protection, combining a high efficacy with a low toxicity for the control of fungal diseases. Within the last 40 years, chemical control has undergone dramatic changes with the detection and introduction of fungicides such as azoles (1969), phenylamides (1977–1983), carboxylic acid amides (CAAs) (1992–2005) and quinone outside inhibitors (QoIs, e.g. azoxystrobin) (1996–2000).^1^ A disadvantage of such single‐site fungicides is their resistance management. As these chemicals often address only one protein within the fungal cell, target mutations will lead to fungicide resistance in most cases. That was intensively studied for the G143A mutation in the cytochrome *b* gene conferring resistance to the QoI fungicides.^2^ However, there have been recurring cycles of introductions of new modes of action, but also losses of efficacy owing to the emergence and selection of resistant pathogen strains.^3^ For a range of pathogen–host combinations, the number of losses of effective fungicides threatens to overcome the number of introductions.^1^, ^4^, ^5^ Thus, resistance management plays a key role in modern plant protection, and there is a growing need to identify new fungicide targets and of course new modes of action. For that reason, so‐called target‐site specific test systems emerged to search for inhibitors of definite targets, i.e. inhibitors of appressorium formation in the rice blast fungus *Magnaporthe oryzae*. For *M. oryzae* and various plant‐pathogenic fungi, appressorium formation is an essential morphological differentiation stage to infect host plants while being dispensable for vegetative growth. Methods to find non‐fungitoxic inhibitors of appressorium formation, DHN‐melanin biosynthesis, spore germination, attachment and penetration of the host surface have been reviewed by Thines *et al.* 6 There is no desperate need for specific targets to be located in fungal life stages involved in the infection‐related morphogenesis. There are also examples of highly successful fungicides addressing single enzymes, signalling cascades or developmental processes that act invariably during fungal life, but they are specific to one pathogen or a close group of pathogens. The phenylpyrrole fludioxonil, the dicarboximide iprodione and the natural product ambruticin are examples of fungicides that interfere with the osmoregulation pathway of filamentous pathogenic fungi. The target protein appears to be a filamentous fungus specific group III hybrid histidine kinase (HK) acting as signal sensor in the HOG pathway.^7^, ^8^, ^9^, ^10^, ^11^ The HOG pathway is composed of a sensory phosphorelay system and a downstream mitogen‐activated protein kinase (MAPK) cascade. Parts of this pathway differ notably in filamentous pathogenic fungi as compared with yeast.^11^, ^12^ The phosphorelay system in the rice blast fungus *M. oryzae* is composed of two HKs, MoSln1p and MoHik1p, the phosphotransfer protein MoYpd1p and the response regulatory protein MoSsk1p.^10^, ^11^, ^13^, ^14^ However, there are ten HK‐encoding genes within the rice blast genome, and it has already been shown that there could be more HKs involved in HOG signalling than MoSln1p and MoHik1p.^15^ The MAPK comprises the MAPK kinase kinase MoSsk2p, the MAPK kinase MoPbs2p and the MAPK MoHog1p. With existing knowledge about *M. oryzae*, it is supposed that under normal environmental conditions the phosphorelay system, more precisely the HKs MoSln1p and MoHik1p, are constitutively active, resulting in constant phosphotransfer via MoYpd1p to MoSsk1p. Thus, there is a high concentration of phosphorylated regulator MoSsk1p. Phosphorylated MoSsk1p is unable to interact with the MAPK kinase kinase MoSsk2p, and therefore the MAPK cascade remains inactive. High external osmolarity results in an inhibition of the sensory HK phosphorylation, and consequently the concentration of phosphorylated MoSsk1p regulator decreases. Dephosphorylated MoSsk1p is able to interact with MoSsk2p, thereby activating the MAPK cascade. Fungicides such as fludioxonil interfere with the phosphorelay system through interaction with MoHik1p and disrupt the phosphorylation pattern responsible for controlled osmoregulation. The regulator MoSsk1p becomes constitutively dephosphorylated, which results in uncontrolled hyperactivation of the MAPK cascade and cell death.^11^ Recently it was shown that the *Magnaporthe* HKs MoSln1p and MoHik1p within the HOG signalling cascade were both individually dispensable for vitality.^15^ MoSln1p appears to be a salt sensor, whereas MoHik1p appears to be a sugar sensor, and both HKs trigger the same pathway components downstream. In contrast to MoHik1p, the HK MoSln1p appears to be involved in pathogenicity, as the *ΔMohik1* mutant strain is much more virulent than the *ΔMosln1* mutant. *ΔMosln1* was found to be almost apathogenic.^15^

Marasmic acid is a sesquiterpenoid with unsaturated dialdehyde functionality and was first isolated from the basidiomycete *Marasmus conigenus* more than half a century ago.^16^ The antibacterial, antifungal, cytotoxic and mutagenic activity was reported previously, and the suggested structure for the broad spectrum of activity was revealed to be the *α*,*β*‐unsaturated aldehyde moiety.^17^, ^18^ However, the detailed biological mode of action of marasmic acid was not elucidated. Toxicity was assumed only regarding effects observed in membrane leakage assays, the AMES *Salmonella* test or an *in vitro* test for inhibition of RNA polymerase II.^17^, ^18^, ^19^ Structure–activity relationships were conducted with marasmic acid and sesquiterpenoid derivatives to understand structural features necessary for biological activity, but the molecular mechanism for the biological activity of marasmic acid has not been clarified in detail to date. One suggestion is that the *α*,*β*‐unsaturated aldehyde reacts with endogenous nucleophiles (e.g. R–SH), and a completely different mechanism has also been suggested in the formation of pyrrole derivatives by the reaction of the two aldehyde groups with endogenous primary amines (R–NH).^18^ However, a distinct mode of action for marasmic acid has not been found to date.

In this study we present marasmic acid interfering with the membrane sensor histidine kinase MoSln1p of *M. oryzae*, thereby hyperactivating the HOG pathway and resulting in cell death. We found that the mode of action of marasmic acid is different to that of the unsaturated dialdehyde sesquiterpenoids merulidial, polygodial, isovelleral and velleral, indicating that the unsaturated dialdehyde moiety in these molecules previously proposed to be required for toxic effects is not involved in antifungal activity in *M. oryzae*.

## 2 EXPERIMENTAL METHODS

### 2.1 Strains and culture/growth conditions

All mutants used in this study were generated from *Magnaporthe oryzae* [*M. oryzae* 70‐15 strain (*MoWT*), Fungal Genetics Stock Centre (FGSC)] in previous studies.^11^, ^15^

The strains were grown at 26 °C on complete medium (CM) [pH 6.5, 2% agar, containing per litre: 10 g of glucose, 1 g of yeast extract, 2 g of peptone, 1 g of casamino acids, 50 mL of nitrate salt solution (containing per litre: 120 g of NaNO_3_, 10.4 g of KCl, 30.4 g of KH_2_PO_4_, 10.4 g of MgSO_4_ · 7H_2_O) and 1 mL of a trace element solution (containing per litre: 22 g of ZnSO_4_ · 7H_2_O, 11 g of H_3_BO_3_, 5 g of MnCl_2_ · 4H_2_O, 5 g of FeSO_4_ · 7H_2_O, 1.7 g of CoCl_2_ · 6H_2_O, 1.6 g of CuSO_4_ · 5H_2_O, 1.5 g of Na_2_MoO_4_ · 2H_2_O, 50 g of Na_2_EDTA, pH 6.5 adjusted by 1 M KOH)].

All chemicals used were p.a. quality unless otherwise stated.

### 2.2 Antifungal compounds

Merulidial and marasmic acid were isolated as described previously and were kindly provided by Prof. T Anke.^18^, ^20^ Isovelleral, velleral and polygodial were kindly provided by Prof. O Sterner.^19^, ^21^, ^22^

### 2.3 Antifungal assays

Antifungal activity was tested with conidia harvested from 11‐day‐old *M. oryzae* cultures and the mutant strains grown on CM. The conidia were filtered through two layers of miracloth tissue (Merck, Darmstadt, Germany) to give a conidial suspension, which was adjusted to 5 × 10^4^ conidia mL^−1^ in H_2_O. Then, the test compounds were added, and the samples were incubated at 26 °C for at least 16 h. The germination and the subsequent initial vegetative growth phase were monitored under the microscope. The concentration resulting in 50% inhibition of conidial germination was defined as IC_50_. The IC_50_ values were calculated by counting the number of germinated conidia out of 100 for each sample. In order to find the IC_50_ range of activity of the tested sesquiterpenoids, we initially conducted a first round of the assay using compound concentrations of 0.1, 1, 5, 10, 20, 50, 100 and 150 µg mL^−1^. Subsequently, we refined the range of compound concentrations for each compound by adding test concentrations very close to the results of the first round (±1 µg mL^−1^). Finally, we conducted assays to evaluate the average IC_50_ values, using five replicates of each compound in the activity‐relevant concentration range.

The antifungal activity of vegetative growth on agar plates was studied using the disc diffusion method. Conidia of the fungal cultures were spread on CM, and filter discs inoculated with marasmic acid were placed in the middle of the plates. Three days post‐application, the zone of inhibition could be evaluated. Five replicates each were used for evaluation.

### 2.4 Western blot analysis of phosphorylated MoHog1p

Phosphorylation of the MAPK MoHog1p in *M. oryzae* was analysed by western blot analysis using an anti Phospho‐p38 MAPK (Thr180/Tyr182) (D3F9) XPTM Rabbit monoclonal antibody (Cell Signaling Technology, Beverly, MA). Total Hog1p was detected using an anti‐Hog1p antibody (Santa Cruz Biotechnology, Santa Cruz, CA). A quantity of 5 mL of CM liquid medium was inoculated with equal amounts of mycelium of *M. oryzae* strains in six‐well cell culture plates (Greiner Bio‐One, Kremsmünster, Austria). After 65 h incubation at 26 °C and 120 rpm, the cultures were exposed to the compounds (25 or 50 µg mL^−1^ for marasmic acid; 10 µg mL^−1^ for fludioxonil) on a shaker for 10 min at room temperature (RT). The cell suspensions were centrifuged at 2900 × *g* for 10 min at 4 °C. The supernatant was discarded, and 300 μL of SDS loading dye (10 mM of Tris‐HCl, pH 6.8, 2.0% SDS, 5% glycerol, 0.1 M of dithiothreitol, 0.01% Bromophenol Blue) was added to the mycelium and heated to 100 °C for 10 min. In order to break the cell walls, glass beads were used in a Ribolyzer Fast Prep FP120 instrument (Thermo Savant, Illkirch, France) for 30 s at 6.0 Hz, followed by a centrifugation step for 5 min at 11 500 × *g*. Equal amounts (25 µg of protein) of the supernatant of the individual samples were separated by SDS polyacrylamide gel electrophoresis and blotted on a nitrocellulose transfer membrane (Roti®‐NC; Carl Roth GmbH, Karlsruhe, Germany) using electrophoretic transfer (Mini Trans‐Blot® electrophoretic transfer cell; Bio‐Rad Laboratories, Munich, Germany). Western immunoblotting was carried out with the Phototope®‐HRP western blot detection system (Cell Signaling Technology) according to the manufacturer's instructions.

## 3 RESULTS

### 3.1 A target‐based in vivo test system to search for inhibitors of the HOG pathway

In order to find new targets within the HOG signalling cascade, a set of mutant strains was generated by means of *Agrobacterium tumefaciens*‐mediated directed gene inactivation. Each mutant was generated in previous studies using a classical gene disruption and gene deletion strategy.^11^ The mutant strains *ΔMosln1*, *ΔMohik1*, *ΔMossk1*, *ΔMossk2*, *ΔMopbs2* and *ΔMohog1* could be used to refer the target location of an antifungal compound directly to the HOG pathway and even narrow down the target protein within this signalling cascade.

Using this target‐based *in vivo* test system, the sesquiterpenoid marasmic acid was found to interfere with the HOG pathway in a different manner as compared with already known fungicides acting in this signalling cascade, e.g. the phenylpyrrole fludioxonil.^11^ Marasmic acid was shown to be antifungal against the *MoWT* in germination assays and in disc diffusion assays, whereas the mutant strains *ΔMosln1*, *ΔMossk1*, *ΔMossk2*, *ΔMopbs2* and *ΔMohog1* were found to be resistant. Interestingly, the mutant strain *ΔMohik1* was not resistant towards marasmic acid (Tables 1 and 2). In contrast, fludioxonil treatment was lethal for the *MoWT* and for the *ΔMosln1* mutant, whereas *ΔMohik1, ΔMossk1*, *ΔMossk2*, *ΔMopbs2* and *ΔMohog1* were resistant (Tables 1 and 2).

**Table 1:** IC_50_ from marasmic acid and fludioxonil in the wild‐type strain and the HOG mutants. The antifungal activity of marasmic acid against the M. oryzae wild type 70‐15 and the HOG mutants was compared with the activity of fludioxonil. Conidia of each strain were harvested, and growth assays were set up in H_2_O as described in the experimental procedures. Differences in the inhibition of different mutant strains are highlighted in grey. The highest concentration tested was 150 µg mL^−1^ for marasmic acid and fludioxonil

*M. oryzae* strain	Marasmic acid (IC_50_ in µg mL^−1^)	Fludioxonil (IC_50_ in µg mL^−1^)
*MoWT* 70‐15	10.2 ± 1.5	4.8 ± 1
*ΔMosln1* (MGG_07312)	>150 µg mL^−1^	5.2 ± 1
*ΔMohik1* (MGG_11174)	10.4 ± 1	>150 µg mL^−1^
*ΔMossk1* (MGG_02897)	>150 µg mL^−1^	>150 µg mL^−1^
*ΔMossk2* (MGG_00183)	>150 µg mL^−1^	>150 µg mL^−1^
*ΔMopbs2* (MGG_10268)	>150 µg mL^−1^	>150 µg mL^−1^
*ΔMohog1* (MGG_01822)	>150 µg mL^−1^	>150 µg mL^−1^

**Table 2:** Inhibition zones of marasmic acid and fludioxonil on vegetatively grown wild‐type cultures and the HOG mutants on solid medium. The antifungal activity of marasmic acid against the M. oryzae wild type 70‐15 and the HOG mutants was compared with the activity of fludioxonil. Disc diffusion assays were set up on CM as described in the experimental procedures. Differences in the inhibition of different mutant strains are highlighted in grey. Three replicates were evaluated

*M. oryzae* strain	50 µg of marasmic acid (inhibition zone in mm)	10 µg of fludioxonil (inhibition zone in mm)
*MoWT* 70‐15	20 ± 2	40 ± 3
*ΔMosln1* (MGG_07312)	None	42 ± 2
*ΔMohik1* (MGG_11174)	22 ± 1	None
*ΔMossk1* (MGG_02897)	None	None
*ΔMossk2* (MGG_00183)	None	None
*ΔMopbs2* (MGG_10268)	None	None
*ΔMohog1* (MGG_01822)	None	None

### 3.2 Western blot analysis confirmed that marasmic acid activates the HOG pathway

**Figure 1:**
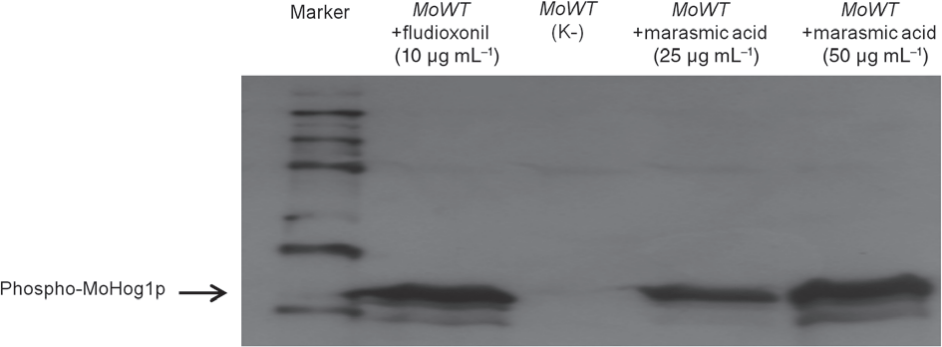
Phosphorylation of the MoHog1 MAPK in M. oryzae after marasmic acid treatment. Mycelium of strain M. oryzae (MoWT) was used to conduct a western analysis as described in the experimental procedures. Incubation in CM was used as negative control (K‐). Marker is a biotinylated protein ladder.

**Figure 2:**
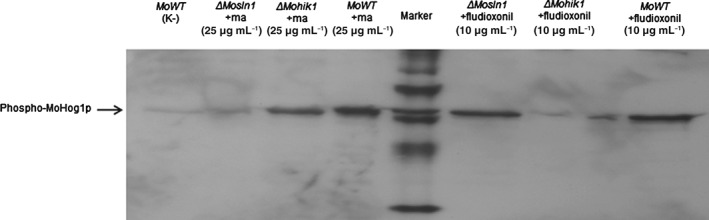
Differences in the phosphorylation patterns of the MoHog1 MAPK in ΔMosln1 and ΔMohik1 after marasmic acid and fludioxonil treatment. Western analysis was conducted as described in the experimental methods. Incubation in CM was used as negative control (K‐) for the MoWT and each mutant strain. As representative for each negative control, we present only the MoWT probe. Marker is a biotinylated protein ladder.

As the results concerning the resistance of mutant strains with an inactivated HOG pathway towards marasmic acid indicated that a signalling cascade, notably the target protein MoSln1p, is required for antifungal activity, we conducted western blot analysis of phosphorylated MoHog1p to detect MoHog1p activation under marasmic acid treatment. Western blot experiments resulted in a dose‐dependent phosphorylation signal after application of marasmic acid in the *MoWT* (Fig. 1). The signal of fludioxonil was used as control of the experiment, as treatment of *M. oryzae* with the phenylpyrrole is known to cause distinct MoHog1p phosphorylation.^11^, ^23^ Additional experiments using the mutant strains *ΔMosln1* and *ΔMohik1* indicated the difference in the putative target proteins of marasmic acid and fludioxonil. Marasmic acid treatment resulted in a strong phosphorylation signal in the *MoWT* and the *ΔMohik1* mutant, whereas fludioxonil treatment resulted in distinct phosphorylation signals in the *MoWT* and the *ΔMosln1* mutant (Fig. 2).

### 3.3 Marasmic acid acts differently to other unsaturated dialdehyde sesquiterpenoids

**Figure 3:**
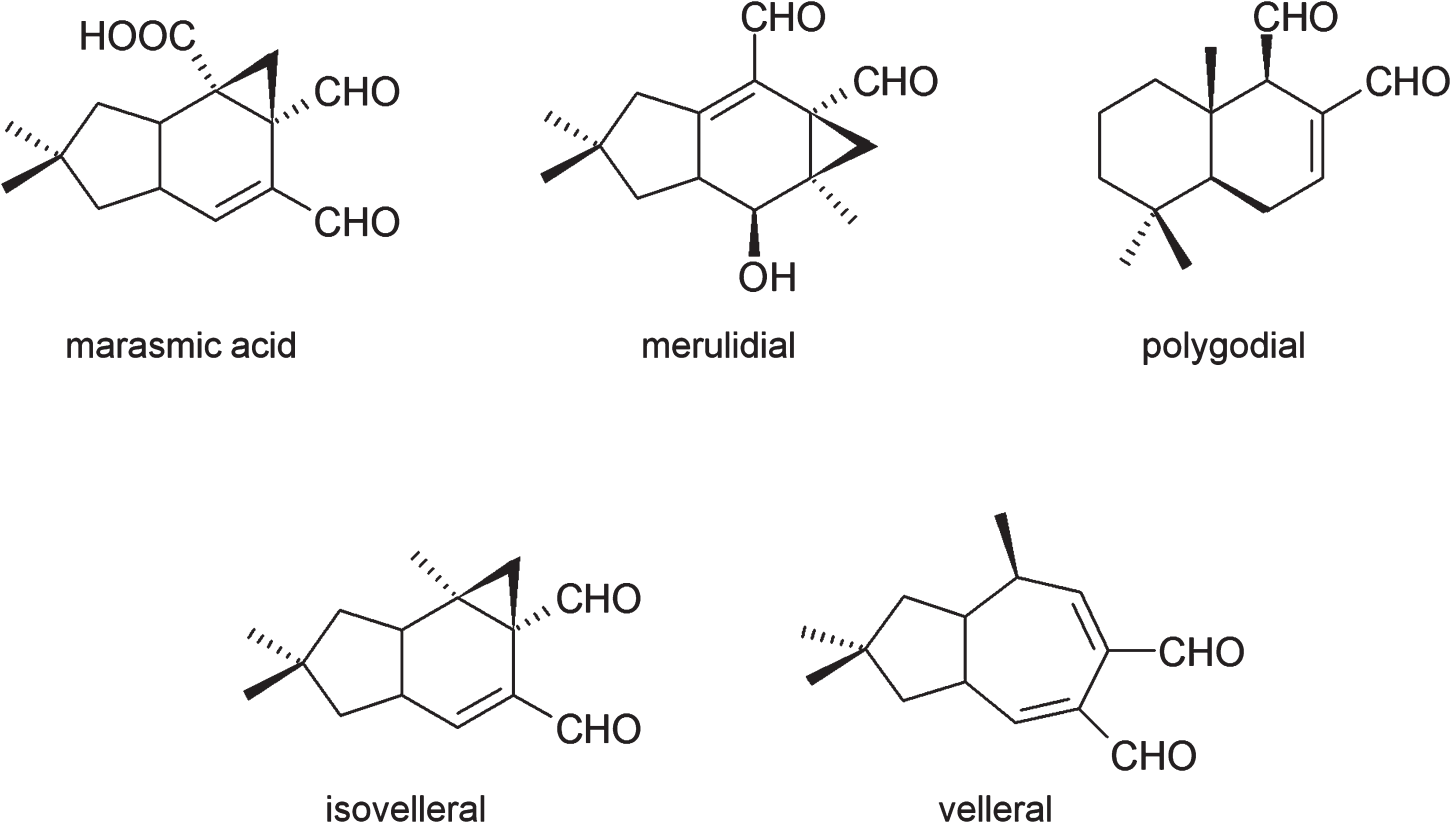
The unsaturated dialdehyde sesquiterpenoids used in this study. Structures of marasmic acid, merulidial, polygodial, isovelleral and velleral.

In order to obtain more details about the structural features of marasmic acid responsible for MoSln1p interaction, we tested a set of sesquiterpenoid derivatives for their activity towards the *MoWT* strain and the mutants with an inactivated HOG signalling cascade. It was proposed before that the activity of unsaturated dialdehydes depends on their accumulation in lipophilic parts of cells (e.g. membranes), their chemical reactivity towards SH groups of proteins associated with membranes and the way they may orient themselves within membrane structures.^17^ As we found our mutant strains to be resistant to marasmic acid, we further tested a set of unsaturated dialdehyde compounds. We compared the antifungal activity of the sesquiterpenoids merulidial, polygodial, isovelleral and velleral with that of marasmic acid (Fig. 3 and Table 3).

**Table 3:** Comparison of the IC_50_ values of unsaturated dialdehyde sequiterpenoid derivatives in the wild‐type strain and the HOG mutants. The antifungal activity of merulidial, polygodial, isovelleral and velleral against the M. oryzae wild type 70‐15 and the HOG mutants was compared with the activity of marasmic acid. Conidia of each strain were harvested, and growth assays were set up in H_2_O as described in the experimental methods. The highest fungicide concentration in the assays was 150 µg mL^−1^

M. oryzae	Marasmic acid (IC_50_ in µg mL^−1^)	Merulidial (IC_50_ in µg mL^−1^)	Polygodial (IC_50_ in µg mL^−1^)	Isovelleral (IC_50_ in µg mL^−1^)	Velleral (IC_50_ in µg mL^−1^)
MoWT 70‐15	10.2 ± 1	24.6 ± 2	5.2 ± 1	2.6 ± 0.5	2.5 ± 0
ΔMosln1 (MGG_07312)	>150 µg mL^−1^	25.4 ± 1.5	5.0 ± 0.5	2.4 ± 0.25	2.7 ± 0.5
ΔMohik1 (MGG_11174)	10.2 ± 1	25.4 ± 1	5.0 ± 0	2.5 ± 0	2.5 ± 0.25
ΔMossk1 (MGG_02897)	>150 µg mL^−1^	25.0 ± 0	5.0 ± 0	2.5 ± 0.25	2.5 ± 0.5
ΔMossk2 (MGG_00183)	>150 µg mL^−1^	25.4 ± 2	4.8 ± 1	2.6 ± 0.5	2.4 ± 1
ΔMopbs2 (MGG_10268)	>150 µg mL^−1^	24.6 ± 2.5	5.2 ± 1	2.5 ± 0.5	2.5 ± 0.5
ΔMohog1 (MGG_01822)	>150 µg mL^−1^	25.2 ± 1	5.0 ± 0	2.5 ± 1	2.4 ± 0.5

In the antifungal assays we found the mode of action of marasmic acid to be different from the mode of action of the other tested unsaturated dialdehydes, as marasmic acid was the sole sesquiterpenoid without activity towards M. oryzae mutants with an inactivated HOG pathway (Table 3). In contrast, merulidial, polygodial, isovelleral and velleral treatment resulted in cell death of M. oryzae and all tested mutant strains.

## 4 DISCUSSION

A variety of technologies in fungicide research to combat fungal pathogens have been developed in the last decades and originated different molecules such as imidazoles, triazoles and morpholines.^24^ A target‐based in vivo test system with previously generated mutant strains in the rice blast fungus M. oryzae can be easily used in order to find inhibitors of proteins in the HOG pathway.^11^ Initial results revealed a novel mode of action for marasmic acid within the HOG pathway of M. oryzae, which is different to known HOG inhibitors, e.g. fludioxonil. The sesquiterpenoid marasmic acid was found to hyperactivate the HOG pathway via the HK MoSln1p, resulting in cell death, whereas M. oryzae mutants with inactivated HOG signalling are resistant to the compound.

**Figure 4:**
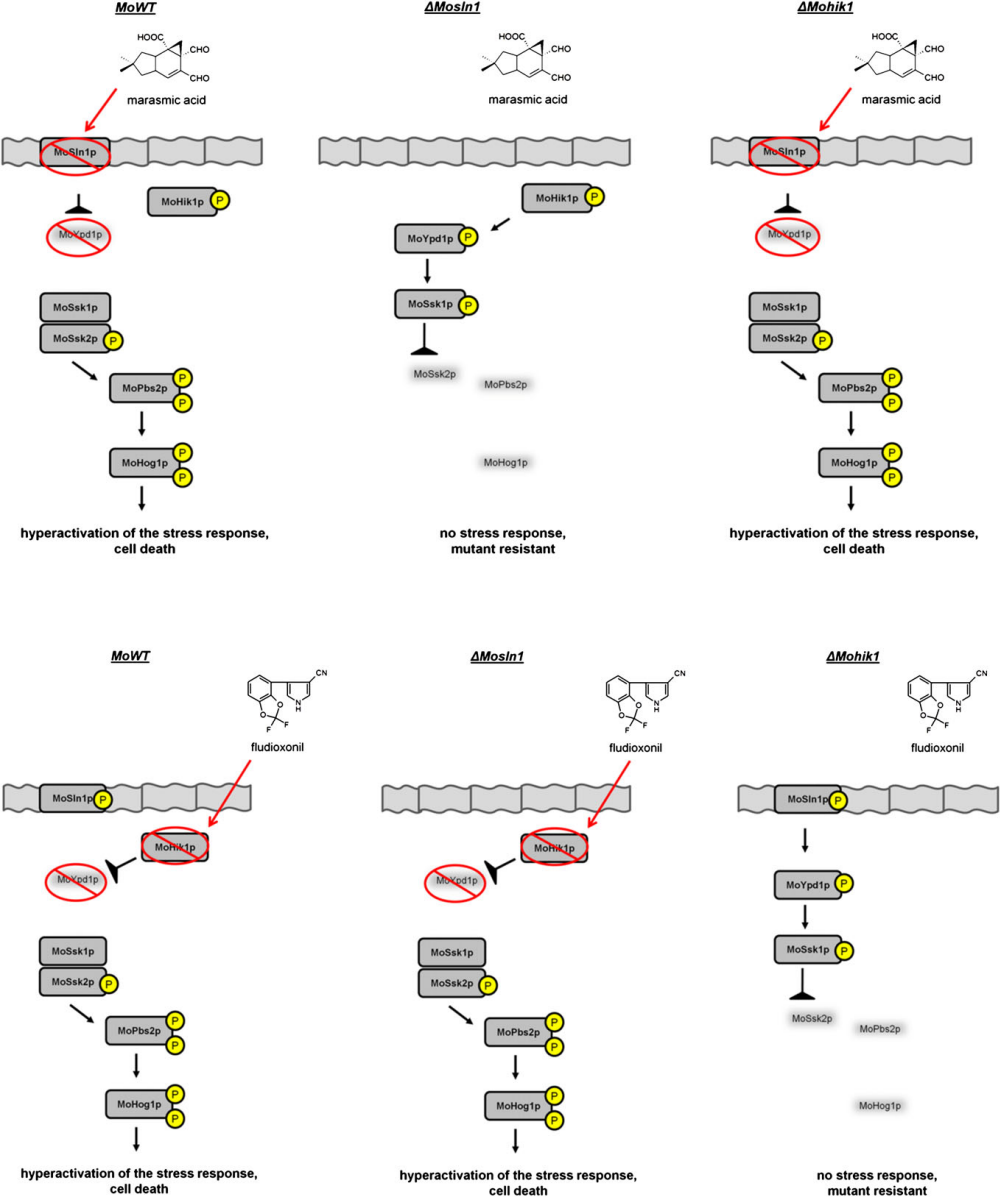
Schematic presentation of the mode of action of marasmic acid and fludioxonil in M. oryzae. The mode of action of marasmic acid and fludioxonil in the wild‐type strain (MoWT) compared with the mutant strains ΔMosln1 and ΔMohik1 is presented. Marasmic acid treatment of the MoWT results in dephosphorylation of the phosphorelay because of interaction with MoSln1p. Therefore, the MAPK cascade MoSsk2p–MoPbs2p–MoHog1p is hyperactivated. Marasmic acid treatment of the ΔMosln1 mutant has no effect, and the phosphorylation patterns in the phosphorelay can persist adequately by functional MoHik1p. Marasmic acid treatment of the ΔMohik1 mutant has equal effects compared with the MoWT. Fludioxonil treatment of the MoWT results in dephosphorylation based on interaction with MoHik1p. For marasmic acid treatment, the MAPK cascade MoSsk2p–MoPbs2p–MoHog1p is hyperactivated. Fludioxonil treatment of the ΔMohik1 mutant has no effect, and the phosphorylation patterns in the phosphorelay can persist adequately by functional MoSln1p. Fludioxonil treatment of the ΔMosln1 mutant has equal effects compared with the MoWT.

Terpenoid unsaturated dialdehydes such as marasmic acid have long been known to be natural defence compounds, and have been shown to possess various potent biological responses, e.g. antifeedant, antibiotic and cytotoxic activities.^25^, ^26^, ^27^ As these toxic effects were assumed obviously to be linked with unsaturated dialdehyde functionality, it was surprising that marasmic acid was found to interfere exclusively with the membrane‐bound sensor HK protein MoSln1p of the HOG pathway of M. oryzae. We clearly documented with our mutant‐based in vivo assays and with additional western blot analysis of MoHog1p phosphorylation that various cytotoxic and mutagenic activities previously assumed for marasmic acid were not prevalent in the filamentous fungus M. oryzae (Tables 1 and 2 and Figs 1 and 2). The mutant strains of M. oryzae with inactivated osmoregulation used in this study were resistant against marasmic acid, thereby indicating that the HOG pathway is indispensable for antifungal activity. Previously published data showed marasmic acid to have the lowest ability to induce membrane leakage among 15 tested sesquiterpenoid derivatives, and the effect of marasmic acid on nucleic acid metabolim was also very low compared with the standard α‐amanitin.^17^, ^18^ The mutagenic activity observed in AMES Salmonella assays was very high, but this assay is a bacterium‐based biological assay for assessing the mutagenic potential of chemical compounds.^19^ Salmonella are prokaryotes, and therefore this assay is not a perfect model for quantifying mutagenic activity of chemicals against fungi or mammals. Altogether, it is reasonable that our findings in this study represent a new mode of action for marasmic acid in the filamentous fungus M. oryzae. To gain a better understanding of the differences to fludioxonil, we present a scheme of the HOG signalling cascade under fungicide treatment in the MoWT strain compared with the effect of both compounds in the ΔMosln1 and the ΔMohik1 mutants (Fig. 4). Both compounds cause a hyperactivation of the HOG pathway, leading to cell death. However, their mode of action appears to depend on interference with the HOG pathway via different proteins. Their interference with the HOG signalling cascade is based on either the sensor HK MoHik1p (fludioxonil) or the sensor HK MoSln1p (marasmic acid), probably resulting in a strong decrease in phosphorylated MoSsk1p regulator. As dephosphorylated MoSsk1p regulator is able to activate the MAPK cascade MoSsk2p–MoPbs2p–MoHog1p, a hyperactivation of MoHog1p is initiated, resulting in cell death. The respective mutant strain without the target proteins MoHik1p (for fludioxonil) or MoSln1p (for marasmic acid) is resistant to the fungicides, as shown by the antifungal assays (Figs 2 and 4 and Tables 1 and 2).

These results are in accordance with previously published data about the different functions of the homologous histidine kinases MoSln1p and MoHik1p in the osmoregulation pathway. It was reported that both HKs are signal sensors contributing to the HOG pathway, with MoSln1p acting as a salt sensor and MoHik1p acting as a sugar sensor.^15^ Thus, it is obvious that antifungal compounds hyperactivating the Hog1p MAPK may have different proteins involved in the mode of action upstream within the HOG pathway.

The activity of unsaturated dialdehydes with a high dipole moment, like the sesquiterpenoid derivatives used in this study, may be based on their accumulation in lipophilic structures of membranes and their tendency to position themselves there as surfactants. The dialdehyde functionality, which is the polar part of the molecule, is believed to be exposed out of the membrane and therefore can react with amino or thiol groups of proteins associated with the membrane.^17^ Thus, marasmic acid may accumulate in the membrane of *M. oryzae* and interfere with MoSln1p. The HK MoSln1p is known to be a transmembrane protein, whereas for MoHik1p no transmembrane domain could be identified. The mutant strain *ΔMosln1* is strongly affected in cell wall stability, which may also be a feature of membrane variance, underlining the important role of MoSln1p in cellular physiology; combined with its important role in pathogenicity, the significance of MoSln1p as a new fungicide target is quite naturally greater.^15^ Further studies involving *in vitro* assays of protein–compound interaction should be conducted, using microscale thermophoresis (NanoTemper Technologies, Munich, Germany) or surface plasmon resonance spectroscopy to find the direct target protein of marasmic acid. The effect of marasmic acid on the whole cellular proteome should also be addressed in the future by detailed phosphoproteome analysis comparing the wild‐type strain and different mutant strains under marasmic acid treatment.

It is known that small structural changes in unsaturated dialdehydes result in considerable variations in activity, as several structure–activity relationships have been performed for these compounds.^28^ We found that the tested unsaturated dialdehydes merulidial, polygodial, isovelleral and velleral appear to act in a different manner to marasmic acid. They are highly fungitoxic towards *M. oryzae*, even the mutants with inactivated HOG signalling (Table 3). Consequently, previous assertions in the literature that unsaturated dialdehyde functionality is the only feature responsible for various strong celltoxic and mutagenic effects cannot explain the mode of action of marasmic acid in the filamentous phytopathogenic fungus *M. oryzae*.^27^ Marasmic acid appears not to promote the suggested reactions for unsaturated dialdehydes with primary amino groups to form pyrrole derivatives or react with endogenous nucleophiles, e.g. R–SH functionalities. Therefore, on the basis of the results of the present study, we postulate a novel mode of action for marasmic acid in the filamentous fungus *M. oryzae*. Marasmic acid hyperactivates the MAPK MoHog1p via MoSln1p within the HOG pathway in a different manner to the known HOG inhibitor fludioxonil.
